# Pemphigoid Antibodies in Patients with Oral Lichen Planus: A Systematic Review

**DOI:** 10.3390/pathophysiology32040051

**Published:** 2025-09-28

**Authors:** Domenico De Falco, Dario Di Stasio, Alessandra Caggiula, Carlo Lajolo, Alberta Lucchese, Massimo Petruzzi

**Affiliations:** 1 Interdisciplinary Department of Medicine, University of Bari “Aldo Moro”, 70124 Bari, Italy; caggiula.alessandra@gmail.com (A.C.); massimo.petruzzi@uniba.it (M.P.); 2 Multidisciplinary Department of Medical and Dental Specialties, University of Campania—“Luigi Vanvitelli”, via De Crecchio, 6, 80138 Napkes, Italy; dario.distasio@unicampania.it (D.D.S.); alberta.lucchese@unicampania.it (A.L.); 3 Head and Neck Department, Fondazione Policlinico Universitario A. Gemelli-IRCCS, School of Dentistry, Università Cattolica del Sacro Cuore, 00168 Rome, Italy; carlo.lajolo@unicatt.it

**Keywords:** Lichen Planus Pemphigoid, LPP, OLP, Oral Lichen Planus, pemphigoid, epitope spreading

## Abstract

**Background/Objectives**: Lichen Planus Pemphigoides (LPP) represents a rare variant of Oral Lichen Planus in which the typical pemphigoid-associated antibodies, BP180 and BP230, are present. The objectives of this Systematic Review are to analyze the data currently available in the literature on this rare condition, with the aim of laying the groundwork for future investigations and research. **Methods**: This Systematic Review was registered in the International Prospective Register of Systematic Reviews (PROSPERO) under the registration number CRD420251133018. Subsequently, a search was conducted on PubMed/Medline, Scopus, and Ovid using specific keywords combined with Boolean operators. Articles published up to 2025 were included. The following types of studies were considered eligible: case reports, clinical conferences, clinical studies, clinical trials, controlled clinical trials, letters, multicenter studies, observational studies, randomized controlled trials, and human-based studies. Book chapters, systematic reviews, narrative reviews, in vitro studies, and animal models were excluded. **Results**: A total of 67 articles were initially identified; following thorough review and exclusion, 20 articles were retained. The patient data extracted from these selected studies were used to construct a table in which patients were categorized according to both qualitative and quantitative variables. The results highlight that LPP is a condition requiring a complex diagnostic process involving both histological examination and serological testing (Immunofluorescence and Enzyme-Linked Immunosorbent Assay—ELISA). **Conclusions**: Furthermore, with the advent of immunotherapy, an increasingly well-documented new category of drug-induced LPP has emerged, associated with PD-1 and PD-L1 inhibitors.

## 1. Introduction

Lichen Planus Pemphigoides (LPP) is a rare variant of Lichen Planus (LP), characterized by the coexistence of clinical and pathological features consistent with Oral Lichen Planus (OLP), together with the presence of autoantibodies against BP180 and BP230, which are typically associated with pemphigoid diseases [1]. The association between OLP and Mucous Membrane Pemphigoid (MMP) was first described by Kaposi in 1892. Subsequently, this condition has been reported in association with neoplasms, viral diseases, phototherapy, and certain types of medications [2]. The prevalence of LPP is approximately 1 case per 1,000,000 patients; however, reported cases are very limited and often underdiagnosed [3]. The sex ratio is skewed toward women, who are more frequently affected [4]. The age of onset is variable, ranging from the third to the sixth decade of life [1]. Clinically, LPP manifests with pruritic erythematous skin lesions accompanied by bullous eruptions. Oral involvement is not always observed; however, when present, it is characterized by erosive and ulcerative lesions developing on pre-existing OLP.

From a clinical, histopathological, and immunoserological standpoint, the diagnosis of LPP may be challenging, as it overlaps with other conditions such as Bullous OLP, Bullous Pemphigoid (BP), and Paraneoplastic Pemphigus (PNP) [1,5,6]. PNP is a rare complication in which an underlying neoplasm triggers immune dysregulation that drives the disease manifestations [6]. Clinically, it differs from related conditions because the lesions are very severe, extensive, and refractory to therapy [6]. It usually regresses following treatment of the underlying neoplasm [6]. Bullous OLP is characterized by the development of bullous lesions on pre-existing lichenoid lesions, whereas in LPP, vesicles typically arise outside the areas of LP involvement (although cases of LPP with vesicle formation on lichenoid lesions have also been reported in the literature) [1]. The vesicles in BP tend to evolve into erosive–ulcerative lesions with a longer and more severe course than those observed in LPP [1]. BP typically affects older patients compared to LPP. LPP lesions more frequently involve the flexural surfaces of the limbs, whereas BP lesions are more generalized [1]. It is believed that LPP arises from the lichenoid inflammation itself, which may promote the development of an autoimmune response against basement membrane proteins through an epitope spreading mechanism—a process also described in the association between OLP and PNP [1,7]. In fact, chronic tissue damage leads to the release of multiple autoantigens, thereby sustaining the immune response against them [2,7,8]. The disruption of the Basement Membrane Zone (BMZ) due to mast cell degranulation may represent the triggering factor for epitope spreading [2,8]. Several cases of drug-induced LPP have been reported in the literature, associated with agents such as angiotensin-converting enzyme inhibitors, programmed cell death protein-1 (PD-1) inhibitors and its ligand (PD-L1) inhibitors including pembrolizumab and nivolumab, as well as drugs such as gabapentin and risankizumab [9,10,11]. The treatment of LPP is largely experience-based and typically begins with corticosteroids, high-potency topical agents for limited disease, and systemic corticosteroids for more extensive or rapidly progressive involvement [1,8,12]. Steroid-sparing options commonly used include dapsone and acitretin [1]. In refractory cases, calcineurin-inhibiting or other immunosuppressive therapies such as cyclosporine or mycophenolate may be considered, and low-dose methotrexate is occasionally employed [1]. More recently, advanced therapies including dupilumab, intravenous immunoglobulin, and rituximab have been reported to induce remission in difficult or drug-induced presentations [12,13,14]. Because LPP is rare and heterogeneous, management should be individualized, with careful evaluation and withdrawal of potential trigger medications when feasible and close monitoring for relapse [1].

The aim of this systematic review is to analyze the documented cases of LPP reported in the literature, in order to shed light on a condition that remains poorly studied and understood, and to provide a basis for future research.

## 2. Materials and Methods

This study was registered on 26 August 2025, in the International Prospective Register of Systematic Review (PROSPERO) under the registration number CRD420251133018. This Systematic Review was conducted in accordance with the Preferred Reporting Items for Systematic Reviews and Meta-Analyses (PRISMA) (Table S1).

### 2.1. Eligibility Criteria

For this Systematic Review, we included case reports, clinical conferences, clinical studies, clinical trials, controlled clinical trials, letters, multicenter studies, observational studies, randomized controlled trials, and human-based studies, while excluding book chapters, systematic reviews, reviews, in vitro studies, and animal models. Furthermore, only studies published in English were considered. The study population included patients with oral manifestations of LPP according with the authors of each article selected.

P (Population): Patients with oral manifestations of LPP, as defined by the authors of the included studies.

I (Intervention): Clinical–histological features compatible with OLP plus immuno-serological evidence of a pemphigoid disorder (autoantibodies to BP180/NC16A and/or BP230 assessed by DIF, IIF, and/or ELISA). Recording of potential drug triggers and treatments administered.

C (Comparison): Not Required.

O (Outcomes): Oral disease features (type, extent, and localization of lesions), involvement of skin and/or other mucous membranes, presence of underlying conditions or pharmacological triggers, serological confirmation (DIF/IIF/ELISA; BP180/NC16A, BP230), Patient outcomes (complete or near-complete remission, persistent disease, relapse).

S (Study design).

- Included: case reports, case series, observational studies, clinical trials, clinical conferences, letters, and other human-based studies.
- Excluded: book chapters, systematic/narrative reviews, in vitro studies, and animal models.

### 2.2. Information Sources and Search Strategy

The literature research covered articles published in English between 1990 and August 2025. The research was performed using MEDLINE/Pubmed, Ovid and Scopus, applying search filters, such as (“oral lichen planus” OR “lichen planus” OR “OLP”) AND (“autoantibodies” OR “antibodies” OR “immunoglobulin G”) AND (“BP180” OR “BP230” OR “NC16A”) AND (“pemphigoid” OR “lichen planus pemphigoides” OR “mucous membrane pemphigoid” OR “autoimmune blistering disease”).

### 2.3. Selection Process

**Figure 1:**
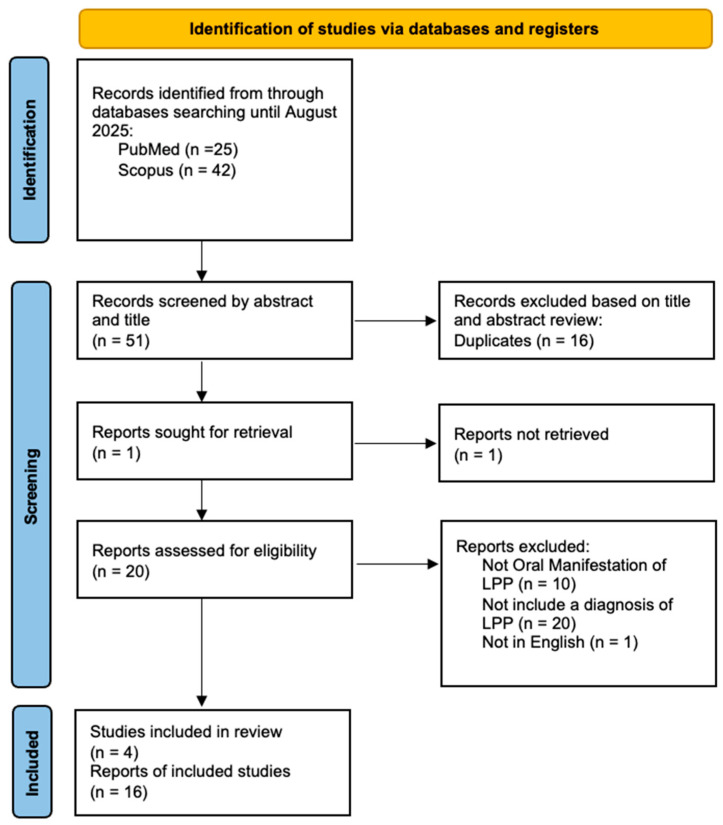
Prisma flow chart.

Two independent authors (D.D.F. and D.D.S.) screened articles by title and abstract for inclusion in the full-text stage. The full text of all potentially relevant articles was examined according to eligibility criteria. Duplicate references across different databases were identified and removed using Zotero version 7.0.26 (Vienna, VA, USA). Disagreements during the full text review process were primarily resolved through discussion between the reviewers. If consensus could not be reached, an independent third reviewer (M.P.) arbitrated the dispute. A flowchart depicting the study selection process is represented in Figure 1.

### 2.4. Data Collection Process and Data Items

Extracted data were independently collected by the reviewers (D.D.F. and D.D.S.) using a Microsoft Excel spreadsheet. From each eligible study, the reviewers (D.D.F. and D.D.S.) extracted data on the first author name and year of publication, as well as age/sex, order of diagnosis (between clinical features of LP and serological findings of Pemphigoid), cutaneous clinical manifestations or involvement of other mucous membranes, localization of oral lesions, direct immunofluorescence (DIF) or indirect immunofluorescence (IIF), Enzyme-Linked Immunoassay (ELISA) testing and clinical outcomes.

### 2.5. Study Risk of Bias Assessment

The risk of bias in the included studies was evaluated using the CASP (Critical Appraisal Skills Programme) tools (Table S2). CASP was selected for its capacity to systematically assess methodological quality and potential bias across different study designs, ensuring a standardized evaluation of methodological rigor.

### 2.6. Effect of Measures

The primary outcome was the clinical manifestations related to oral LPP. These included the extent of oral lesions, involvement of other tissues (skin, other mucous membranes), the presence or absence of underlying conditions whose pharmacological treatment might have triggered LPP, serological confirmation of the clinical conditions, and patient outcomes.

### 2.7. Synthesis Methods

A descriptive, qualitative analysis was carried out on the extracted data. Categorical variables were summarized using frequencies and percentages, while qualitative findings were presented through a narrative synthesis. Since the evidence was limited to case series and case reports, performing a meta-analysis was not possible.

## 3. Results

### 3.1. Study Selection

A total of 67 articles were identified, of which 16 were duplicates, 20 did not include a diagnoses of LPP, 10 did not report oral manifestations of LPP, and 1 was excluded because it was not in English. articles were identified through manual search. The data from the remaining 20 articles were included in a table (Table 1) based on variables such as age/sex, order of diagnosis (between clinical features of LP and serological findings of Pemphigoid), cutaneous clinical manifestations or involvement of other mucous membranes, localization of oral lesions, direct immunofluorescence (DIF) or indirect immunofluorescence (IIF), Enzyme-Linked Immunoassay (ELISA) testing and clinical outcomes.

### 3.2. Study Characteristic

The study evaluated 13 case reports, 4 retrospective studies, and 3 case series. The included studies were published between 1999 and 2025. Among patients with LPP, the mean age was 60.3 years (range 34–86). Sex was reported in 36 patients, of whom 11 (30.6%) were male and 25 (69.4%) female. With regard to the timing of onset between the typical clinicopathological features of LPP and those of pemphigoid, in 22 cases (61.1%) they occurred simultaneously, in 11 cases (30.6%) LPP preceded Autoimmune Bullous Disease (AIBD), and in 3 cases (8.3%) AIBD preceded LPP. Oral involvement was relatively homogeneous across clinical presentations, with oral erosions reported in 21 cases (42%), oral ulcers in 21 cases (42%), oral mucositis in 2 cases (4%), and other manifestations in 4 cases. Cutaneous involvement was observed in 27 patients (56%), combined cutaneous and other mucosal involvement in 8 patients (16.7%), exclusive involvement of other mucosal sites in 6 patients (12.6%), and isolated oral involvement in 7 patients (14.6%).

Eighteen patients (38%) with LPP were receiving medications potentially associated with the onset of lesions. Among these, 11 cases (56%) were related to oncologic immunotherapy, while the remaining 44% were associated with antihypertensive agents, statins, antidiabetic drugs, antivirals, and psychotropic medications. In the remaining 32 patients (62%), no evidence of drug-related triggering factors was identified. Regarding diagnostic investigations, direct immunofluorescence (DIF) was positive in 36 patients (72%), with C3-only deposition detected in 3 patients (6%). Indirect immunofluorescence (IIF) was positive in seven cases (14%). Immunoblotting revealed anti-BP180 antibodies in 13 patients (26%), anti-BP230 antibodies in 2 patients (4%), and other targets in 1 patient (2%). ELISA testing detected anti-BP180 antibodies in 34 patients (68%) and anti-BP230 antibodies in 6 patients (12%). As for patient outcomes, 21 patients (58.4%) achieved near-complete remission following pharmacologic treatment, 10 patients (27.8%) achieved complete remission, while in 3 patients (8.3%) the disease remained active.

## 4. Discussion

LPP is a heterogeneous disorder with features overlapping those of OLP and MMP [3,4]. Diagnosis is often challenging, as it requires histopathological findings consistent with OLP in combination with serological evidence of MMP [4]. Our systematic review indicates the presence of two distinct categories of LPP: drug-induced and non–drug-induced. Among diagnostic and serological tests, DIF and ELISA emerged as the most sensitive modalities. Corticosteroid therapy was effective in the majority of cases. In drug-induced LPP, withdrawal of the triggering medication, together with the aforementioned therapeutic approach, was consistently associated with clinical improvement.

The data reported in our study are consistent with the findings of previous individual studies on LPP. In fact, LPP is confirmed to be a condition affecting a wide age range, from young adults (around 30 years old) to individuals in the sixth and seventh decades of life [4]. As reported in the literature, women are more frequently affected than men [8]. Most authors describe synchronous oral lichen planus–like lesions associated with pemphigoid [8]. However, some authors report cases in which OLP represents the initial manifestation, with subsequent unmasking of LPP [8]. Remarkably, Mignona et al. even described two cases with the reverse sequence, where MMP evolved into LPP [2]. Another element fully in line with other studies is the consistent coexistence of oral and cutaneous lesions, which are almost invariably present [4]. Indeed, in the general case series of LPP, more than half of the patients show exclusively cutaneous involvement [4]. The involvement of mucous membranes, particularly the oral mucosa, is observed in a subset of patients (<50%) with LPP [4,8].

With regard to oral clinical manifestations, these are fairly homogeneous and not pathognomonic, as they consist of ulcerative–erosive lesions also observed in other clinical conditions such as pemphigus, OLP, pemphigoid, paraneoplastic pemphigus, and other oral mucositides [6,8].

Several studies have shown an association between drug exposure and LPP; however, effects upon drug re-exposure have not been documented, and further clinical validation is therefore required [1]. Although cases of drug-induced LPP, such as those triggered by ACE inhibitors, are well documented in the literature, with the advent of immunotherapy the proportion of drug-related cases is expected to increase [11,15,28]. Therefore, future studies should investigate the risk of LPP following immunotherapy in larger cohorts [9,15]. The diagnosis of LPP represents one of the most debated issues in the literature on this condition, due to its histological overlap with OLP and serological overlap with MMP [8]. Our study, in line with the literature, emphasizes that serology—particularly ELISA testing—is decisive for the diagnostic confirmation of this disease. Finally, treatment, which involves the use of systemic corticosteroids, immunosuppressants, IVIg, monoclonal antibodies, and, in drug-induced cases, discontinuation of the causative agent, appears to yield at least partially favorable outcomes [8].

## 5. Conclusions

The present study is intended as an initial step toward consolidating the available case studies of LPP, with the aim of facilitating a more precise diagnostic framework for this condition in the future. Nevertheless, it is subject to certain limitations, primarily related to the restricted amount of data and the predominance of case reports and case series within the current literature. In conclusion, we believe, in agreement with other authors, that due to its diagnostic complexity and heterogeneous clinical presentation, LPP is likely an underdiagnosed condition [8,29]. For this reason, in order to advance scientifically in the development and understanding of this disease, large multicenter studies are needed.

## Figures and Tables

**Table 1:** This table summarizes all documented cases of Oral Lichen Planus Pemphigoides according to the established search criteria.

Study ID	Patients No.	Age/Sex	Order of Diagnosis (LP First/AIBD First)	Skin/Mucosal Findings	Involved Oral Sites	Underlying Condition	DIF/IIF	ELISA (Positive > 9 U/mL or > 20 RU/mL)	Outcomes
**Maeda K et al., J Dent Sci., 2025 [15].**	1	82/M	AIBD first	Skin	Oral Mucositis	PB—Lung Metastasis from Renal Carcinoma	**DIF** (Oral): IgG, C3 on BMZ	Anti BP180 NC16a (366 U/mL)	Improvement after drug discontinuation
**Jadhav A et al., Ann Afr Med., 2025, Apr [3].**	1	42/M	LP first	Skin	Oral Erosion		**DIF** (Skin): IgG, C3	Anti BP180 (59.9 Ru/mL)	CR with OMZ and Betamethasone therapy
**De D, Mustari AP et al., Indian Dermatol Online J., 2025, May 26 [4].**	1			Skin	Oral Ulcer		**DIF** (Skin): IgG, C3	Anti BP180	
	1			Skin	Oral Ulcer		**DIF** (Skin): IgG, C3	Anti BP180	
	1			Skin	Oral Ulcer		**DIF** (Skin): IgG, C3	Anti BP180	
	1			Skin	Oral Ulcer		**DIF** (Skin): IgG, C3	Anti BP180	
	1			Skin	Oral Ulcer		**DIF** (Skin): IgG, C3	Anti BP180	
	1			Skin	Oral Ulcer		**DIF** (Skin): IgG, C3	Anti BP180	
**Wang S et al., J Cutan Pathol., 2024, Feb [11].**	1	53/F	At once		Oral Ulcer: Lips, dorsal tongue, buccal mucosa	Anti-PD1—Stage IV Melanoma	**DIF** (Oral): IgG, C3, IgA		Improvement after drug substitution
**Combemale L et al., Front Immunol., 2024, Apr 15 [8].**	1	86/F	LP first	Skin, Genital	Oral Erosion		**DIF** (Oral): IgG, C3	Anti BP180	CR with DOX and DDS therapy
	1	43/F	LP first	Skin, Nose and throat	Oral Erosion		**DIF** (Oral): IgG, IgA, IgM and C3		aCR with DDS therapy
	1	58/M	LP first	Skin, Genital, Anal	Oral Erosion	HL			CR with DDS therapy
	1	74/F	LP first	Skin, Genital	Oral Erosion		**DIF** (Oral): IgG, C3	Anti BP180	aCR with DDS therapy
	1	82/F	LP first	Skin, Genital, Nose and throat	Oral Erosion		**DIF** (Oral): IgG, IgA	Anti BP180	AD—DDS therapy
	1	69/M	LP first	Genital, Anal	Oral Erosion				AD—DDS and tCS therapy
	1	71/F	LP first	Nose and throat	Oral Erosion				CR with tCS therapy
	1	35/M	LP first	Genital	Oral Erosion			Anti BP180, BP230, Col VII	Controlled with DOX and DDS therapy
	1	58/F	AIBD first	Skin, Nose and throat	Oral Erosion		**DIF** (Oral): IgG, C3		CR with DDS therapy
	1	65/F	AIBD first	Genital, Conjunctiva	Oral Erosion		**DIF** (Oral): C3		CR with DDS therapy
	1	42/F	At once	Skin, Genital	Oral Erosion	NHL—Pembrolizumab	**DIF** (Oral): IgG, C3	Anti BP180	AD—DDS therapy
	1	66/F	At once	Skin, Genital, Anal	Oral Erosion		**DIF** (Oral): IgG, C3	Anti BP180, BP230	aCR with DDS therapy
**Ney ZC, Nicholson LT et al., JAAD Case Rep., 2024, Sep 20 [14]**	1	60/F	At once	Skin	Hemorrhagic-crusted erosion on the lips, erythema and erosion on the palatal and buccal mucosa	PB—Stage IIIB Melanoma	**DIF** (Oral): IgG, IgA and C3 on BMZ **IB**: Anti BP 180 and 230		aCR after PB discontinuation and IVIg—HCQ Therapy
	1	70/M	At once	Skin	Oral Ulcer: Tongue	PB—Metastatic Gastric Adenocarcinoma	**IIF**: IgG on BMZ (BP180, 230)	Anti BP180 and BP230	aCR after PB discontinuation and IVIg- HCQ therapy
**Liu SS et al., Am J Dermatopathol., 2023 Apr [16]**	1	60/M	At once	Skin	Oral Erosion	Nivolumab—Hepatocellular carcinoma	**DIF** (Skin): IgG, C3	Negative	CR after Nivolumab discontinuation and sCS and INX therapy
**Ajaaouani R et al., Cureus. 2022 Nov; [17].**	1	68/F	At once	Skin	Oral Ulcer	Gliclazide—Diabetes type II	**DIF**: IgG, C3		aCR after drug substitution
**Wat M et al., J Cutan Pathol., 2022, Nov [18]**	1	80/F	At once	Skin	Lip and Tongue Erosion	PB- Metastatic Lung Adenocarcinoma	**DIF** (Skin): IgG, C3	Anti BP180 (44 U/mL)	aCR after PB discontinuation and sCS and IVIg therapy
	1	77/M	At once	Skin	Oral Mucositis	PB- NSCLC	**DIF** (Skin): IgG, C3 **DIF** (Oral): C3	Anti BP180 (42.6 U/mL), Anti BP230 (5.83 U/mL)	aCR after PB discontinuation and sCS therapy
**Boyle MM et al., Am J Dermatopathol., 2022, May [10].**	1	66/F	At once	Skin	Oral Erosion: Lips, Tongue, Hard Palate White Lesions: Lip, Tongue, Buccal mucosa, gum, Hard palate	Nivolumab, Sitravatinib Urothelial Carcinoma	**DIF** (Skin): IgG and C3	Anti BP180 (126 RU/mL) Anti BP230 (105 RU/mL)	aCR after Nivolumab discontinuation and RTX and sCS therapy
	1	57/F	At once	Skin	Oral Erosion	PB, Metastatic NSCLC	**DIF** (Skin): IgG, C3 **IIF**: Negative	Anti BP180 (52 RU/mL) Anti BP230 (3 RU/mL)	aCR after PB discontinuation and sCS therapy
**Ondhia, Chandni MRCP et al., The American Journal of Dermatopathology, November 2019 [19].**	1	70/F	At once	Skin	Oral Ulcers: Hard Palate	Perindopril, Bendroflumetiazide- Hypertension	**DIF** (Skin): IgG, C3, Fibrinogen **IIF**: Anti BMZ (IgG)	Anti BP180 (86 U/mL)	aCR after drugs discontinuation and sCS and IVIg therapy
**Jang SH. et al., Clin Exp Dermatol., 2015, Dec [20].**	1	56/F	At once	Skin	Oral Ulcers	Entecavir, Ursodesoxycholic acid- HBV infection	**DIF** (Skin): IgG **IB**: Anti BP 189 and 230 kDa		aCR after sCS and DDS therapy
**Sekiya A et al., Br J Dermatol., 2014, Nov [21].**	1	58/F	At once	Skin	Oral Erosion		**DIF** (Skin): IgG, C3 **IIF**: Anti BMZ (IgG) IB: Anti BP180, BP180 NC16a, 120 kDa LAD-1	Anti DSG1 (370 U mL^−1^), Anti BP180 NC16a domain (93 U mL^−1^)	aCR after sCS and diaphenyl sulfone treatment
**Washio K et al., Case Rep Dermatol., 2013, Mar [22].**	1	35/F	At once	Skin	Oral Erosion: Buccal mucosa		**DIF** (Skin): IgG, C3 on BMZ **IIF**: IgG **IB**: Anti BP180-NC16a domain	Anti BP180 (39 U/mL)	aCR after CyA and sCS treatment
**Mignogna MD et al., Oral Surg Oral Med Oral Pathol Oral Radiol Endod., 2010, Jun [2]**	1	72/F	LP first	Ocular	Oral Erosion	Simvastatin	**DIF** (Oral): IgG on BMZ **IIF**: positive	Anti BP180	CR after sCS and IVIg therapy
	1	64/F	LP first	Ocular, Vaginal		Atorvastatin, sertraline	**DIF** (Oral): IgG on BMZ **IIF**: positive	Anti BP 180	CR after Atorvastatin discontinuation and sCS therapy
**Buijsrogge JJ et al., J Dermatol Sci., 2007, Sep [23].**	1				Oral Ulcer				
	1				Oral Ulcer		**IB**: Anti BP180 (IgG)		
	1				Oral Ulcer		**IB**: Anti BP180 NC16a (IgG)		
	1				Oral Ulcer		**IB**: Anti BP180 NC16a (IgG)		
	1				Oral Ulcer		**IB**: Anti BP180 (IgG)		
	1				Oral Ulcer		**IB**: Anti BP180 (IgG)		
	1				Oral Ulcer		**IB**: Anti BP180 (IgG)		
	1				Oral Ulcer		**IB**: Anti BP180 (IgG)		
**Zhu YI et al., Int J Dermatol., 2006 [24].**	1	69/F	At once	Skin	Oral Ulceration	Ramipril—Hypertension	**DIF**: IgG, C3 on BMZ		CR after drug discontinuation
**Sakuma-Oyama Y et al., Clin Exp Dermatol., 2003, Nov [25].**	1	47/M	At once	Skin	Oral Ulcer	Trimipramin—Depression	**DIF** (Skin): colloid bodies with IgM and IgA on BMZ, C3 **IIF**: C3 at the BMZ	Anti BP180 (31–110 U/mL)	aCR after treatment with IVIg and Mycophenolate mofetil
**Skaria M et al., Dermatology., 1999 [26]**	1	47/M	LP first	Skin		Enalapril, tiludronate—Arterial hypertension and Paget’s disease	**DIF**: C3, along BMZ **IIF**: IgG **IB**: Anti BP180		Patients lost at follow-up
**Zillikens D et al., J Invest Dermatol., 1999, Jul [27]**	1	52/F	At once	Skin	White Streaks on buccal mucosa		**DIF** (Skin): IgG, C3 on BMZ **IB**: Anti BP180 Epidermal, NC16a	Anti BP180 NC16a	aCR after sCS therapy
	1	43/M	At once	Skin	White Streaks on buccal mucosa		**DIF** (Skin): C3 on BMZ **IB**: Anti BP180 Epidermal, NC16a	Anti BP180 NC16a	aCR after sCS therapy
	1	61/F	At once	Skin	White Streaks on buccal mucosa		**DIF** (Skin): IgG, C3 on BMZ **IB**: Anti BP180 Epidermal, NC16a	Anti BP180 NC16a	aCR after tCS therapy
	1	34/F	At once	Skin	White Streaks on buccal mucosa		**DIF** (Skin): IgG, C3 on BMZ **IB**: Anti BP180, NC16a	Anti BP180 NC16a	aCR after sCS therapy

AIBD: Autoimmune Bullous Disease; AD: Active Disease; BMZ: Basement Membrane Zone; CR: Complete Remission; aCR: Almost Complete Remission; tCS: Topical Corticosteroids; sCS: Systemic Corticosteroids; CyA: cyclosporine; DDS: dapsone; DIF: Direct Immunofluorescence; DOX: Doxycycline; ELISA: Enzyme-Linked Immunosorbent Assay; HCQ: Hydroxycloroquine; IIF: indirect immunofluorescence; IB: immuno-blotting; IVIG: intravenous immunoglobulin; INX: infliximab; LP: Lichen Planus; NSLCC: Non Small Lung Cell Carcinoma; OMZ: omalizumab; PB: pembrolizumab; RTX: rituximab.

## Data Availability

The original contributions presented in this study are included in the article/Supplementary Materials. Further inquiries can be directed to the corresponding author.

## Supplementary Materials

The following supporting information can be downloaded at: https://www.mdpi.com/article/10.3390/pathophysiology32040051/s1, Table S1: PRISMA checklist; Table S2: CASP RoB.
